# Information Theory Analysis of CTX Shows Consistent Clinical Presentation

**DOI:** 10.1002/jimd.70098

**Published:** 2025-10-22

**Authors:** Jennifer Hanson, Penelope E. Bonnen

**Affiliations:** ^1^ Department of Molecular and Human Genetics Baylor College of Medicine Houston Texas USA

**Keywords:** bile acids, cerebrotendinous xanthomatosis, CTX, CYP27A1, lipid storage disorder

## Abstract

Cerebrotendinous xanthomatosis (CTX) is a rare, metabolic disorder caused by pathogenic variants in *CYP27A1*. The classic clinical presentation includes infantile‐onset chronic diarrhea, juvenile‐onset bilateral cataracts, with development of tendon xanthomas and progressive neurological dysfunction. These multisystem clinical features typically appear in different decades of life often confounding diagnosis of CTX. Further complicating diagnosis is the generally held belief that the clinical presentation of CTX varies highly between individuals and even within families. We applied information theory analyses to CTX patient data to quantitatively assess clinical variability in CTX. We conducted a systematic review of the literature to identify all CTX families reported with *CYP27A1* genotype (*N* = 218). Information theory analyses of subject data across 12 clinical features of CTX showed a remarkably consistent clinical presentation within families, with just four out of 83 families demonstrating notable phenotypic variability. Further analysis of subjects with two pathogenic missense variants versus two loss of function variants showed higher clinical burden in loss‐of‐function group (*p* = 0.0001). We surmise that the multi‐system, progressive nature of CTX developing across decades leads to variable characterizations of the disease and that standardization of terms and comparison of clinical features within age decade reveals a more consistent clinical presentation. The identification of the common, consistent features of CTX may be useful for screening and diagnosis of this treatable disorder. This study illustrates that information theory analyses can be leveraged to detect clinically relevant information even in the absence of large‐scale datasets, such as is often the case for rare disease.

## 1 Introduction

Cerebrotendinous xanthomatosis (CTX) is an inborn error of metabolism, the hallmark features of which include chronic diarrhea, juvenile‐onset cataracts, tendinous xanthomas, and progressive neurological dysfunction (OMIM 213700). CTX is caused by bi‐allelic pathogenic variants in *CYP27A1*, which encodes for the protein sterol 27‐hydroxylase. Loss of function of sterol 27‐hydroxylase, which is essential for bile acid synthesis, results in decreased bile acid synthesis and elevations in bile acid intermediates, including cholestanol, the elevation of which is often used as a biomarker of CTX. Diagnosis can be made with biochemical testing and/or genetic testing of *CYP27A1*.

While some subjects with CTX experience symptoms from infancy, diagnosis is often not made until the third or fourth decade of life due to the multi‐system clinical presentation and the emergence of features over decades [1, 2]. Many reports assert that CTX clinical presentation is highly variable across individuals, even within families, and attempts to assess genotype–phenotype associations have reported there are none [2, 3, 4, 5, 6]. Treatment with the bile acid chenodeoxycholic acid (CDCA) is available that significantly slows progression of disease, and when started early, can prevent the major clinical problems associated with this disease, underscoring the importance of early diagnosis [1, 7].

In this study, we bring a systematic approach to understanding the clinical presentation of CTX and test if applying an information theory algorithm can bring clarity to the question of the extent of clinical variability between patients with CTX. Hamming distance is a metric of similarity developed at Bell Labs in 1950 for application in error detection and correction in telecommunications data transmission [8]. Since that time, Hamming distance has been utilized in multiple domains, including cryptography, computer science, artificial intelligence, and machine learning, particularly aiding in tasks like pattern recognition and clustering. We apply Hamming distance to clinical data to quantitate the similarity or difference between individuals with CTX.

We conducted a systematic review of the literature and found 92 families with two or more individuals diagnosed with CTX who had both a confirmed genotype and full clinical description. These data were analyzed using Hamming distance based on the most common features of CTX (cataracts, tendon xanthoma, chronic diarrhea, osteopenia/osteoporosis, intellectual disability, dementia, psychiatric disturbance, seizures, cerebellar dysfunction, pyramidal dysfunction, peripheral neuropathy and parkinsonism). This information theory approach to analyzing clinical presentation in a rare disease that is historically reported to be highly variable revealed consistent patterns within families. This study exemplifies that information theory analyses can be leveraged to detect clinically relevant information even in the absence of large‐scale datasets, such as is often the case for rare disease.

## 2 Methods

### 2.1 Literature Search

The study was carried out in accordance with the Preferred Reporting Items for Systematic Reviews of Meta‐Analysis (PRISMA) guidelines [9]. Search of the medical literature was conducted to identify families with more than one individual with confirmed *CYP27A1* pathogenic genotype and clinical details. We systematically reviewed the literature for all cases of CTX published up to June 30, 2025, written in English and contained in the National Institute of Health PubMed and Ovid Medline databases. The search was conducted using the query [title, keywords, and MeSH the terms “cerebrotendinous xanthomatosis”].

### 2.2 Study Selection

Records (*N* = 506) were downloaded to citation software (Endnote 21; Clarivate Analytics) and duplicates were removed automatically. Records were reviewed manually and the remaining duplicates were removed (*N* = 248). Records (*N* = 258) were screened and reports (*N* = 240) were assessed for eligibility (Figure S1). Only peer‐reviewed manuscripts written in English were selected for review. Remaining studies were searched to identify subjects who were diagnosed with CTX, whose *CYP27A1* genotype and clinical case description were published in an individually identifying manner. Studies were excluded if they did not contain a clinical case description for subjects with CTX. Studies were excluded if they did not contain families with at least 2 members diagnosed with CTX, and if their pathogenic genotype in *CYP27A1* was not reported. Finally, studies were excluded if there were no clinical details for at least two family members in an individually identifiable manner. The references in every selected paper were examined for additional studies on the same cases.

### 2.3 Inclusion Criteria for Subjects

Subjects were included in this study if they had a verified *CYP27A1* genotype, a clinical case description reported in an individually identifying manner, and had at least one additional nuclear family member reported who also met these criteria [1, 2, 3, 4, 7, 10, 11, 12, 13, 14, 15, 16, 17, 18, 19, 20, 21, 22, 23, 24, 25, 26, 27, 28, 29, 30, 31, 32, 33, 34, 35, 36, 37, 38, 39, 40, 41, 42, 43, 44, 45, 46, 47, 48, 49, 50, 51, 52, 53, 54, 55, 56, 57, 58, 59, 60, 61, 62, 63, 64, 65, 66, 67, 68, 69, 70, 71, 72, 73, 74, 75, 76, 77, 78, 79, 80, 81, 82, 83, 84, 85, 86, 87, 88, 89, 90, 91, 92, 93, 94, 95, 96, 97, 98, 99, 100, 101, 102, 103, 104, 105, 106, 107, 108, 109, 110, 111, 112, 113, 114, 115, 116, 117, 118, 119, 120, 121, 122, 123, 124, 125, 126]. For subjects that were published in multiple papers, all papers were reviewed and clinical information was extracted in order to compile the fullest clinical case description. In total, 218 subjects diagnosed with CTX were included in this study, and data on these subjects was extracted from 122 published manuscripts.

### 2.4 Data Extraction Process

Two reviewers extracted data independently, with disagreements resolved by consensus in accordance with PRISMA guidelines [9]. When patients were in multiple papers and there were disagreements, the papers were re‐reviewed independently, and a consensus was determined by the reviewers.

The following data were extracted: age at onset, age at diagnosis, age at death, age at last report, cataract, optic disk pallor, tendon xanthomas, osteoporosis, osteopenia, pes cavus, intellectual disability, learning difficulty, developmental delay, cognitive decline, dementia, psychiatric disturbances, seizures, epilepsy, cerebellar signs, dysmetria, nystagmus, dysdiadochokinesia, dysarthria, speech disturbances, ataxia, gait abnormality, sensory ataxia, cerebellar atrophy, dentate nucleus lesions, cerebellar white matter lesions, intention tremor, pyramidal signs, clonus, hypotonia, spasticity, hyperreflexia, Babinski sign, spastic paraparesis, spastic tetraparesis, parkinsonism, resting tremor, bradykinesia, hypokinesia, akinesia, rigidity, masked face, hypomimia, peripheral neuropathy, axonal neuropathy, demyelinating neuropathy, polyneuropathy, diarrhea, neonatal jaundice, cholestasis and cholestanol levels.

Cataracts were selected when individuals were noted as having cataracts or juvenile cataracts. Tendon xanthoma was selected when individuals were noted as having tendon xanthomas on any parts of their body. The osteoporosis/osteopenia category was selected when patients were noted as having osteoporosis or osteopenia or when bone mineral density assessment z‐scores were below the expected range for age. Seizure was selected when patients were reported as having epilepsy or seizures.

Cognition was variably described across publications. Psychiatric disturbance was selected when patients were noted as having a psychiatric disorder or when they were reported to have behavioral changes, anxiety, agitation, aggression, delusions, depression, hallucinations, apathy, or suicide. For this study, intellectual disability was defined as when an individual was reported as having intellectual disability or “mental retardation” under the age of 22. The dementia category was defined as when an individual was noted as having dementia with onset over the age of 21 or with evidence of clinical testing using standardized instruments (MOCA or MMSE).

Cerebellar signs were selected when patients were noted as having “cerebellar signs,” ataxia, MRI abnormality cerebellar atrophy, MRI abnormality in the cerebellar dentate nucleus, and MRI abnormality cerebellar white matter lesions. Pyramidal signs were selected when subjects were noted as having ‘pyramidal signs’, spasticity, clonus, hyperreflexia, Babinski sign, and extensor plantar response. Parkinsonism was selected when subjects were noted as having ‘parkinsonism’ or two or more signs of parkinsonism specifically: resting tremor, bradykinesia, hypokinesia, akinesia, rigidity, masked face, and hypomimia. The peripheral neuropathy category was selected when subjects were reported to have ‘peripheral neuropathy’, polyneuropathy, axonal neuropathy, or demyelinating neuropathy.

Nomenclature for all variants follows HGVS standards and is reported in reference to *CYP27A1* NM_000784.3 and NP_000775.1.

### 2.5 Measuring Clinical Distance Using Hamming Distance

The major clinical features of CTX reported in this study were cataracts, tendon xanthoma, chronic diarrhea, osteopenia/osteoporosis, intellectual disability, dementia, psychiatric disturbance, seizures, cerebellar dysfunction, pyramidal dysfunction, peripheral neuropathy, and parkinsonism. The presence or absence of these features was recorded for each individual. If a feature was not mentioned explicitly, it was recorded as NR (not reported). Dementia was defined as a decline from prior cognitive functioning after the age of 21, and as a result, all subjects under the age of 22 were noted as NA (not available) for dementia. A subject was marked as having a clinical feature if they ever exhibited the feature.

Clinical features of affected individuals were compared in a pairwise fashion and given a distance score by calculating Hamming distance [8]. Hamming distance measures the number of positions at which two strings of equal length have different symbols at corresponding positions in the string. Mathematically, for two strings *x* and *y* of equal length n, the Hamming distance *D* (*x*, *y*) is expressed as:

$$D\;\left(x,y\right)=\sum_{i=1}^{n}1\left(x_{i}\neq y_{i}\right)$$

### 2.6 Genotype Phenotype Association Across Unrelated Individuals

Individuals were subdivided into groups according to genotype. The positive predictive value for each of the 12 most common clinical features of CTX was calculated as the number of individuals with the clinical feature divided by the number of individuals with the genotype of interest. The margin of error was calculated using *t*‐distribution‐based statistics. To assess differences in the means of continuous variables (e.g., age of onset, age at last report), we used Student's *t*‐test. Correlation coefficients were used to evaluate whether two variables tended to change together.

## 3 Results

### 3.1 CTX Families Description

**FIGURE 1:**
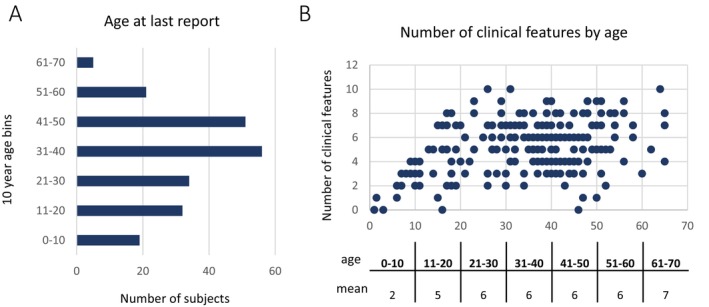
Age at last report by decade for the 218 individuals with CTX in this study. (A) Subjects were grouped by decade for age at last report, and the number of subjects was plotted for each decade. (B) The number of clinical features each person exhibited was reported on the *Y* axis and plotted according to their age on the *X* axis. The mean number of clinical features in subjects who fell into each decade of life at last report is shown below the scatter plot.

Two hundred and eighteen subjects diagnosed with CTX comprising 92 families were identified through systematic review of the medical literature. The age distribution for age at last report was 1 year to 65 years old, with most subjects in their 30's and 40's (Figure 1). CTX is a progressive disorder that evolves across decades. The precise decade of onset for each clinical feature is not known definitively and is another likely source of variability in disease presentation. For example, many individuals with CTX may develop ataxia in the third decade of life while others may not develop ataxia until later. Before measuring clinical variability within families, we examined how age may influence the presence or absence of a clinical feature.

In order to better understand, and subsequently control for, the effects of age on the clinical presentation of this progressive disease, we assessed the number of clinical features present in each individual in the study (*N* = 218). This was plotted according to age, and the average number of clinical features present in subjects in each decade of life was calculated (Figure 1B). The average number of clinical features present in the first decade of life was 2; it was 5 in the second decade and then remained stable at 6 from the 20's until the 60's. As a result, subjects younger than 11 years old were excluded from the phenotypic variability distance analysis. There were a remaining 199 subjects across 83 families. Most families in this study were sibpairs (*N* = 55); there were also 23 sibships with 3 siblings and 5 sibships with 4 siblings.

Review of systems for each subject in this group of families revealed the most common features reported were cataracts, tendon xanthoma, chronic diarrhea, osteopenia/osteoporosis, intellectual disability, dementia, psychiatric disturbance, seizures, cerebellar dysfunction, pyramidal dysfunction, peripheral neuropathy, and parkinsonism (Table S1).

**FIGURE 2:**
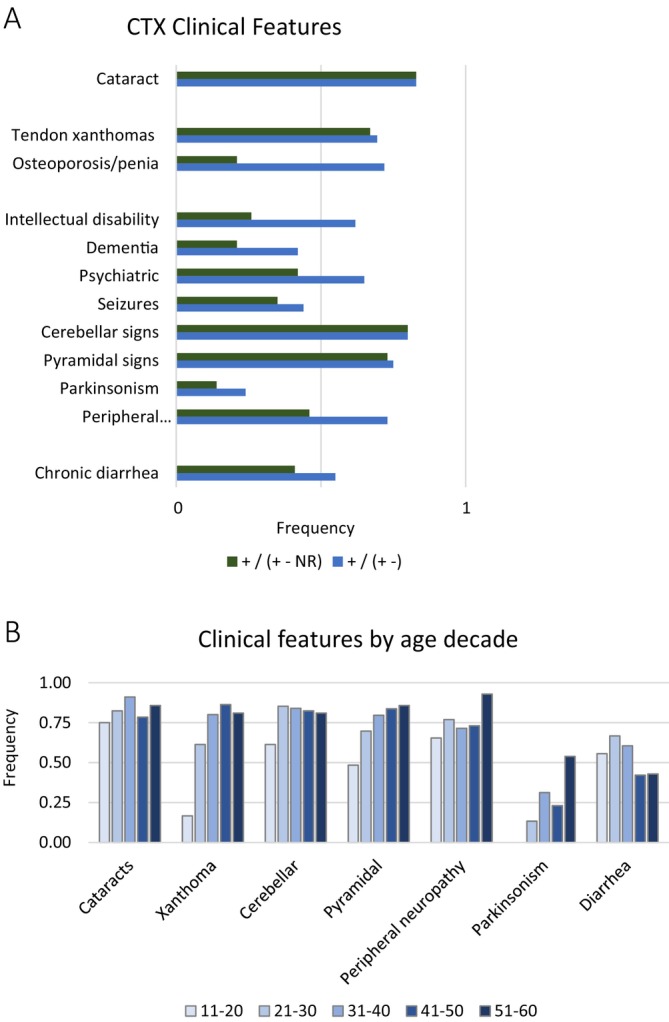
Frequency of the most commonly occurring CTX clinical features in this study. (A) The frequency of each clinical feature was determined for the group of individuals in this study who were older than 10 years old. Frequency was calculated in two ways in order to illustrate the extent of missing data in the cohort for each clinical feature. Frequency was calculated as the number of subjects who had a feature divided by the total number of subjects (green). It was also calculated as the number of individuals reported to have the feature divided by the number of individuals who were reported explicitly have or not have the feature (blue). (B) the frequency of individual clinical features by age decade. Individuals were grouped according to the age at last report and the frequency of the clinical feature in each decade is shown. Frequency was calculated as the number of subjects who had the feature divided by total number of individuals in the age decade.

The frequency of cataracts in the subjects in this group of families who were above the age of 10 years at last report was 83%, which is similar in range to that previously reported in other cohorts [1, 2, 127] (Figure 2). The most prevalent musculoskeletal system feature was tendon xanthomas, which occurred in 69% of subjects (Figure 2). Osteopenia/osteoporosis had a high frequency in the subjects who were evaluated for bone density (72%); however, this feature was not reported in most subjects and, as a result, the frequency of osteopenia/osteoporosis was 21% in the overall cohort (Figure 2). Chronic diarrhea was reported for 55% of individuals.

Every subject over the age of 19 experienced some neurological problem, and these fell into the main categories of: intellectual disability, dementia, psychiatric disturbances, seizures, cerebellar dysfunction, pyramidal dysfunction, parkinsonism, and peripheral neuropathy.

In individuals with CTX, cognitive deficits may appear in childhood or adulthood, and depending on the age of onset and severity, they will be described differently. The term intellectual disability (ID) is defined in the DSM‐5 as significant cognitive deficits resulting in lowered ability in intellectual functioning and adaptive behaviors, with an age of onset beginning in childhood. In contrast, dementia, which was named major neurocognitive disorder (NCD) in the DSM‐5, is characterized by acquired cognitive deficits in adulthood, which represent a significant decline from the prior level of functioning, rather than cognitive deficits present from childhood. The frequency of subjects who had ID was 26% (62% of individuals for whom ID was reported or excluded), and those who had dementia was 21% (42% of individuals for whom dementia was reported or excluded) (Figure 2). Psychiatric disturbance, which refers to behavioral manifestations such as agitation, aggression, delusions, hallucinations, depression, and apathy, was reported in 42% of subjects (65% of subjects for whom this was reported).

Seventy‐nine percent of individuals displayed signs of cerebellar dysfunction, with just 1% of subjects not reported (Figure 2) (Table S1). The pyramidal tract is comprised of the corticospinal tract and corticobulbar tracts. Damage to the corticospinal tract can present as spasticity, clonus, hyperreflexia, and Babinski sign, and spastic paraparesis. Damage to the corticobulbar tract can present as pseudobulbar palsy. In this study, 73% of individuals showed signs of pyramidal tract dysfunction, all of which were signs of corticospinal tract dysfunction.

Peripheral neuropathy, including axonal, demyelinating, and polyneuropathy, was reported in 46% of subjects, but was present in 73% of individuals in whom it was evaluated (Figure 2). Parkinsonism was reported in 13% of the cohort. The age range of these individuals was 38–65 years, and the prevalence of parkinsonism in individuals in this study who were 38 or older was 26%. Seizures were also reported in 35% of subjects (Figure 2).

The frequency of clinical features was also assessed per age decade (Figure 2). Xanthoma showed a consistent increase in frequency from 11 years to 50 years. Pyramidal signs showed a similar trend. Parkinsonism did not appear until the 30s, which is categorized as early‐onset parkinsonism. Cataracts appeared in the second decade of life and increased marginally in frequency after that. One drawback of analyzing the data in this way is that this frequency at age data was not longitudinal within individuals as the age of onset of each feature was not available. This representation of the data may yield insight into the age of onset of features, but it does not replace longitudinal studies of individuals.

### 3.2 Family‐Based Clinical Distance Study

The major clinical features of CTX reported in this study were cataracts, tendon xanthoma, chronic diarrhea, osteopenia/osteoporosis, intellectual disability, dementia, psychiatric disturbance, seizures, cerebellar dysfunction, pyramidal dysfunction, peripheral neuropathy, and parkinsonism. For each individual, it was noted whether they ever experienced each of these features (Table S1). The extent of clinical variability between individuals was measured using Hamming distance. This quantifies differences between two entities in a pairwise manner. In this case, it measures clinical variability between individuals; each clinical feature that was discordant between two individuals was counted, and each pair received a distance score of 0–12, with 0 indicating that both members of the pair exhibited the same clinical features and 12 indicating that both members of the pair were discordant for all 12 of the clinical features under consideration.

**FIGURE 3:**
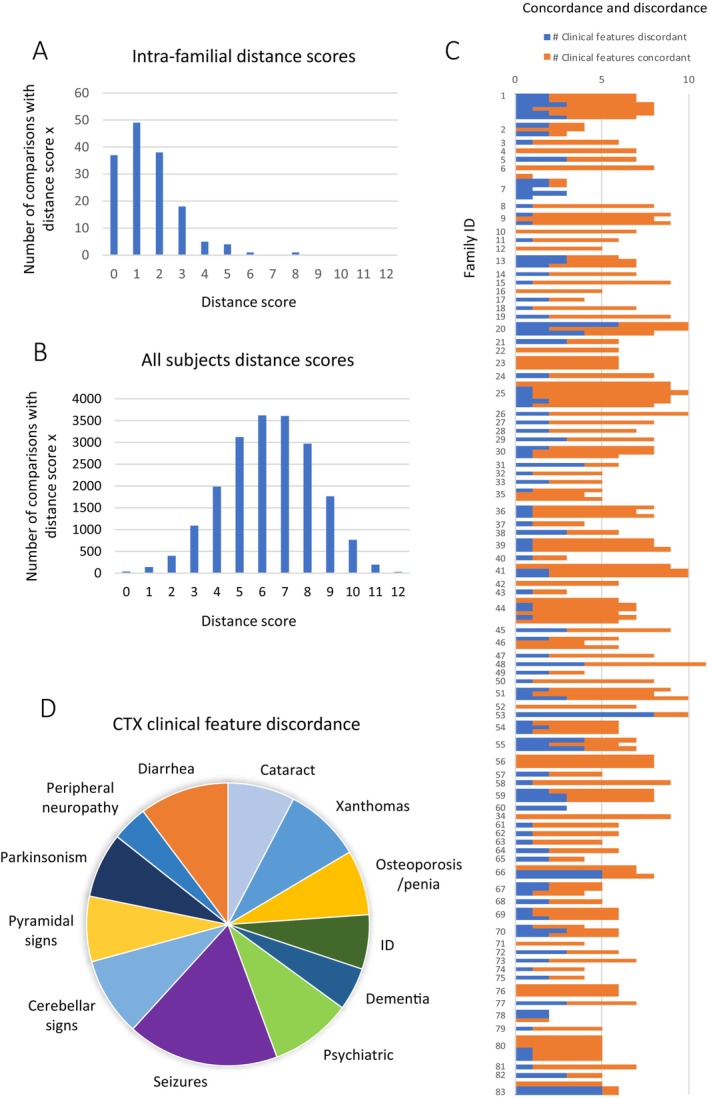
Distance scores from intra‐familial pairwise comparisons show consistency in clinical presentation within families. The 12 most common CTX clinical features in this cohort were compared in affected individuals in the same family in a pairwise fashion and Hamming distance was calculated for subjects over the age of 10. (A) The number of intrafamilial CTX affected pairs that had a distance score (Hamming distance) for each possible distance score was plotted. (B) Hamming distance was calculated for all possible pairs of individuals in this study. There were 199 subjects over the age of ten and the distance scores from resulting from the 19 701 unique pairwise comparisons are plotted on the *Y* axis. (C) For each pairwise intra‐familial comparison the number of clinical features that were concordant are shown in orange and the number of clinical features that were discordant were shown in blue. Pairwise comparisons from the same family were plotted together and family ID is shown on the *Y* axis. (D) The rate that a clinical feature was discordant across all pairwise comparisons.

Each individual in a family was compared to the other individuals in the same family in a pairwise manner. We conducted 154 comparisons within 83 families. The results of this intra‐family pairwise comparison study showed consistency in clinical presentation within most families (Figure 3A). As a control experiment, Hamming distance was calculated between all subjects in the study (number of unique pairwise comparisons = 19 701) (Figure 3B). The distribution of distance scores computed between all subjects was a normal distribution. The distribution of distance scores for intrafamilial comparisons was not normal.

Thirty‐seven pairs were completely concordant in their clinical presentation (Figure 3A). The most common Hamming distance score was 1, meaning these pairs had one clinical feature for which they were discordant. The next most common distance score was 2; 38 pairs had two discordant features. We also asked if there was a connection between the number of clinical features displayed in a family and the distance within the family. The number of concordant clinical features was plotted with the number of discordant features (Hamming distance) for each pairwise comparison and grouped by family (Figure 3C). There was no relationship detected between the total number of clinical features exhibited in a family and the number of clinical features that were discordant in intra‐family pairwise comparisons, indicating that the number of discordant features did not strongly depend on the total number of clinical features within families (r^2^ = 0.2).

There were a small number of families that exhibited greater variability in clinical presentation between family members. One sibpair has a distance score of 8 and was discordant for every clinical feature reported, despite being 53 and 44 years old at last report. This data was reported as part of a large cohort and there was no narrative provided for these subjects, giving no further insights. One family comprised of three siblings affected with CTX had ages at last report of 60, 64, and 65; they were close in age and old enough to have experienced the full range of CTX clinical features (Table S1, Family 20) [31]. One sibling experienced the most clinical features and had cataracts, xanthomas, chronic diarrhea, ID, dementia, psychiatric disturbances, seizures, cerebellar dysfunction, pyramidal dysfunction, and parkinsonism. In contrast, the index case in the family experienced four of these clinical features, yielding a distance score of 6. The third sibling experienced eight of the same clinical features as the more severe case, yielding a distance score of 2 and a distance score of 4 with the index case. There were two families who showed a similar pattern of distance (Families 66 and 83). They were both comprised of three affected siblings and both had distance scores of 0, 5, 5 indicating that one member of the family was different from the other two who were completely concordant. In one family, the ages were 58, 54, and 45; the siblings closer in age were concordant and the youngest sibling has fewer clinical features than the older two. Family 83 was similar with two older siblings aged 35, 32, and one younger who was 18. In both families, age may play a role in the discordance and there remains the possibility that the younger siblings' clinical presentation will progress to include more overlap.

Some clinical features were discordant within families more often than others (Figure 3C). Seizure was the most frequently discordant feature within families, with 35% of pairs discordant. Most other features showed a similar extent of discordance ranging from 10% (dementia) to 20% (diarrhea). The feature that showed the smallest amount of discordance between pairs was peripheral neuropathy (8%).

### 3.3 Assessment of Missing Data and Its Effect on Hamming Distance

Clinical features were reported for each individual in the study when they were explicitly stated in the original report/s for subjects as being present or absent. When they were not clearly stated as present or absent, NR was entered into the clinical data table (Table S1). Multiple sources of missing data were considered. The frequency that each clinical feature was NR was calculated (Figure S2A). Missing not at random (MNAR) was apparent for osteoporosis/osteopenia, as most studies did not attend to this less well‐appreciated clinical feature of CTX (NR = 70%). The clinical sign with the second most frequent amount of missing data was intellectual disability (NR = 58%). This is a more widely appreciated feature of CTX; we speculate that the delay in the diagnosis of CTX may contribute to intellectual disability since this is a feature of childhood. A missingness map was created that showed each subject per row roughly ordered by year of publication (Figure S2B). This order was not precise due to multiple subjects having been published multiple times in different years. However, the missingness map shows a trend toward older studies, those published prior to 2005, having more missing data than more recent studies.

Hamming distance conducted in a pairwise manner within families was unaffected by these sources of MNAR since data for families was reported in the same original study. If a study did not report a feature for any individuals in the study, this MNAR did not affect the Hamming distance score, as the NR would be a match across all subjects in the study. To test how other sources of missingness may affect Hamming distance, particularly missingness completely at random (MCAR), we computed Hamming distance with NR as a match and then again with NR as a mismatch. There was no correlation between the number of NR and Hamming distance (*r* ^2^ = 0.05). In addition, the distribution of Hamming distance scores calculated with NR counted as a mismatch versus the distribution of distance scores calculated with NR not counted as a mismatch was not different (Student's *t*‐test *p* value = 1). We concluded that Hamming distance is robust to multiple sources of missingness in this dataset.

### 3.4 Genotype Phenotype Analysis

**FIGURE 4:**
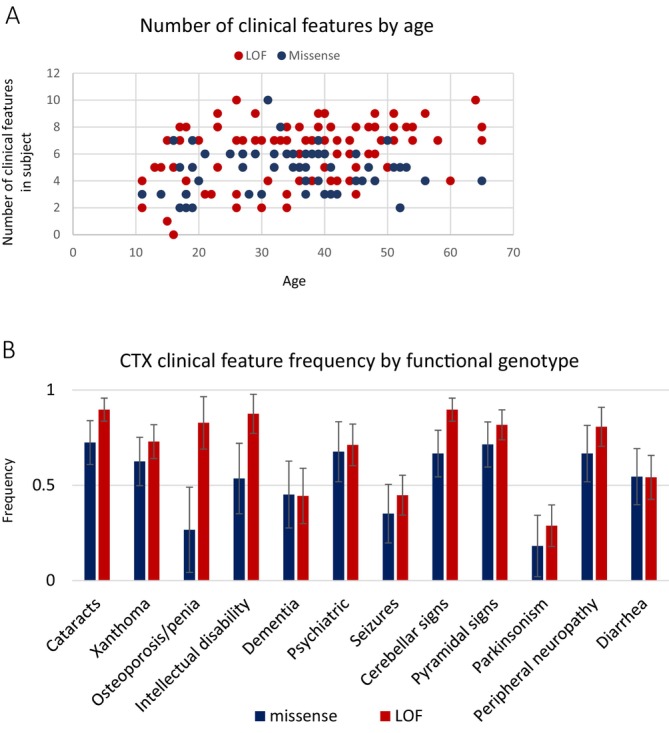
Genotype phenotype analysis. (A) The number of clinical features by age was plotted for the subjects who had two pathogenic missense variants in *CYP27A1* (*N* = 42, blue) and the subjects who had two LOF variants in *CYP27A1* (*N* = 97, red). (B) Comparison of the frequency of CTX clinical features by genotype. The frequency of each of the 12 most common CTX clinical features was calculated and plotted for each genotype group (LOF vs. missense) along with the margin of error for the positive predictive value of genotype to each individual clinical feature.

Prior attempts to discern genotype–phenotype associations in *CYP27A1* and CTX failed to detect any associations [2, 3, 4, 5, 6]. The sample sizes in these studies were small, as is often the case in rare disease, and individual genotypes were tested, which divided the sample into even smaller groups for testing. We took an approach to maximizing sample size to the extent possible and grouped subjects according to ‘functional genotype’. In addition, since the average number of clinical features present in the first decade of life was lower than in all other decades (Figure 1B), subjects younger than 11 years old were excluded from the genotype–phenotype analysis, just as they were excluded from the phenotypic variability distance analysis. Subjects over the age of 10 who had two loss of function (nonsense, splice, frameshift) variants in *CYP27A1* were binned into one group (*N* = 97); subjects over the age of 10 who had two missense variants in *CYP27A1* were binned into another (*N* = 42). The age distribution between these two groups was not different (*p* value = 0.67). The number of clinical features in each individual was plotted by age (Figure 4). The distribution of the number of clinical features was higher in the group of subjects with LOF variants than in the group with missense (*p* value = 0.0001). We next asked if the distribution of the age of onset was different between the LOF and missense groups, and there was no statistical evidence of any difference (*p* value = 0.4).

A genotype–phenotype association study was conducted by comparing the 12 most common clinical features of CTX in individuals with two LOF variants versus individuals who had two missense variants (Figure 4). This study design shed insight into whether having complete loss of function of sterol 27‐hydroxylase might result in different clinical features than having two missense or potentially partial functioning variants in *CYP27A1*. The results did not show statistically significant differences between the two genotype groups.

## 4 Discussion

Systematic review of familial CTX cases resulted in the characterization of the clinical and genetic features of a group of 199 individuals comprising 83 families. Previous reports of the variability in clinical presentation between individuals with CTX reported high variation in clinical presentation across patients with CTX, including in families [2, 3, 4, 5, 6]. We applied an information theory algorithm, Hamming distance, to test the hypothesis that there are clinical signs of CTX that appear across patients consistently enough to facilitate diagnosis. We showed that measuring the distance between patients by examining the presence/absence of clinical signs reveals more similarity than differences. Most families showed consistency in clinical presentation, with 56% of intra‐familial pairwise comparisons showing zero or one clinical feature as discordant.

We compared the result of our information theory‐based analysis on specific families that had previously been reported as having large phenotypic differences between patients. A set of twins included in this study scored a 2 in our approach to measuring clinical discordance. These 40‐year‐old twins were previously reported as having ‘different phenotypes’ of CTX [4]. Both twins had cataracts, cerebellar signs, parkinsonism, dementia, and psychiatric disturbances. One twin was additionally reported as having an ‘isolated, pronounced episode of diarrhea’, the most highly variable clinical feature in this study, and pyramidal signs of brisk patellar reflex and ankle clonus on the right side with bilateral Babinski sign. These identical twins experienced a highly overlapping constellation of clinical features, including parkinsonism, which is one of the less common features of CTX. However, the severity of these features was more debilitating in one twin, resulting in real differences in quality of life and required supportive care. Similarly, an additional 12 families analyzed here were previously published in manuscripts that characterized CTX as having high intra‐familial variability [2, 3]. The distance score when measured by our analysis was most often 1. This analysis illustrates that when clinical presentation is characterized and compared in a systematic, quantitative manner, a pattern can be discerned among family members with CTX in most families.

Genotype‐phenotype studies of CTX are difficult to conduct with sound statistical power due to the rarity of the disease and many pathogenic variants. Instead of comparing specific genotypes, we compared individuals who had two LOF variants versus those who had two missense variants. Comparison of the frequency of each of the 12 most common clinical features of CTX between individuals who possessed two LOF variants versus individuals who had two missense variants did not show significant differences between the two genotype groups. However, the mean of the distribution of the number of clinical features was higher in the group of subjects with LOF variants than in the group with missense (*p* value = 0.0001). These results indicate a higher disease burden for individuals with CTX caused by two LOF variants.

Variation in the frequency of clinical features of CTX has also been reported at the cohort level. Cataracts and cerebellar signs were the two most common features of CTX in this study at 81% and 80%, respectively, of the subjects over the age of 10. Pyramidal signs (63%), peripheral neuropathy (59%), xanthomas (55%), chronic diarrhea (52%), and psychiatric disturbances (50%) were the next most frequent features in this cohort. Comparison of the prevalence of clinical features in this study with those in other previously reported cohorts shows some large differences in reporting (Table S2). For example, one of the hallmark signs of CTX, tendon xanthomas, was diagnosed in 55% of individuals in this study over the age of 10, while they were reported at a prevalence of 78% [2], 34% [1], and 77% [127] in other CTX cohorts. Over nearly all clinical features of CTX, there were differences in the reported frequency of clinical features across various cohorts. This could be due to differences in the range of age at last report of subjects and/or may indicate various types of ascertainment bias. This could also be influenced by the fact that CTX is a multi‐system disorder requiring the attention of multiple medical specialists in order to fully characterize an individual's clinical presentation.

In the course of this study, we considered testing for association between cholestanol values and clinical presentation. However, there are some barriers to using cholestanol in association testing. One major challenge to conducting a genotype‐biochemical phenotype analysis with cholestanol is that various labs around the world use different methods to measure the cholestanol levels, and they use different reference ranges. In addition, cholestanol measurements were conducted at different ages and stages of disease progression, and many were reported without noting the age of the patient, severity of the disease, or stage of the disease. In addition, the patients for whom we found multiple measurements showed variation in cholestanol levels, and no explanation for this variation was given. For all of these reasons, we did not have the data necessary to test for association with cholestanol.

Severity of disease was not considered in measuring concordance; rather, strictly the presence or absence of the 12 most frequent clinical features observed in this study. Severity of disease was not quantified due to patients being reported in the literature by many different groups and having received varying instruments and descriptors for assessing their disease presentation. This study does not intend to minimize the impact and importance of severity of disease on individuals and their care, but rather to identify the commonalities in CTX disease course in an effort to better identify and support future patients and their families. Studies have shown that early treatment with CDCA can prevent progression of the disease and increase cognition scores in CTX patients [1, 16, 45, 119], a strong motivation for early diagnosis and treatment.

## Author Contributions

Penelope E. Bonnen conceived and designed the study as well as analyzed and interpreted data. P.E.B. wrote the article. Jennifer Hanson extracted all of the data from the literature, created data tables, and helped draft the article.

## Ethics Statement

This article does not contain any studies with human or animal subjects performed by any of the authors.

## Conflicts of Interest

The authors declare no conflicts of interest.

## Supporting information


**Table S1:** Clinical findings in subjects with CTX.

**Table S2:** Frequency of clinical features reported across different CTX cohorts.

**Figure S1:** jimd70098‐sup‐0003‐Figures.pdf.
**Figure S2:** jimd70098‐sup‐0003‐Figures.pdf.

## Data Availability

All data used in this study is publicly available in articles indexed in PubMed.
