# Supplementary material for: Information Theory Analysis of CTX Shows Consistent Clinical Presentation
Source: J Inherit Metab Dis. 2025 Oct 22;48(6):e70098. doi: 10.1002/jimd.70098 (PMC12541572; doi:10.1002/jimd.70098)
Supplement: Supplementary file 2 — Table S2: Frequency of clinical features reported across different CTX cohorts. [file JIMD-48-0-s002.docx]

Supplemental table 2. Frequency of clinical features reported across different CTX cohorts.

|  | This Study | Mignarri 2014 | Duell 2018 | Stelten 2019 |
| --- | --- | --- | --- | --- |
| Cataracts | 83% | 89% | 70% | 88% |
| Tendon xanthomas | 67% | 78% | 77% | 34% |
| Chronic Diarrhea | 41% | 40% | 53% | 70% |
| Seizures | 35% | 33% | NR | 7% |
| Cerebellar signs | 80% | 36% | NR | 39% |
| Pyramidal signs | 73% | 64% | NR | 45% |
| Osteoporosis | 21% | 67% | NR | NR |

1. Mignarri, A., et al., *A suspicion index for early diagnosis and treatment of cerebrotendinous xanthomatosis.* J Inherit Metab Dis, 2014. **37**(3): p. 421-9.

2. Stelten, B.M.L., et al., *Long-term treatment effect in cerebrotendinous xanthomatosis depends on age at treatment start.* Neurology, 2019. **92**(2): p. e83-e95.

3. Duell, P.B., et al., *Diagnosis, treatment, and clinical outcomes in 43 cases with cerebrotendinous xanthomatosis.* J Clin Lipidol, 2018. **12**(5): p. 1169-1178.

4. Verrips, A., et al., *Clinical and molecular genetic characteristics of patients with cerebrotendinous xanthomatosis.* Brain, 2000. **123 ( Pt 5)**: p. 908-19.

Comparison of the prevalence of clinical features in this study to other previously reported cohorts shows some large differences in reporting. The frequency shown in this table indicates the number of individuals in this study over the age of 10 who had the clinical feature out of the total number of individuals in this study over the age of ten (N = 199, >10). The frequency of cataracts in the subjects in this study who were above the age of 10 years at last report was 83%, which is lower than the 88-90% previously reported in some other cohorts [1, 2] but higher than the 70% reported in another [3]. No subjects in this study less than 10 years of age were reported as having cataracts, suggesting that this clinical features of CTX may not appear in the first decade of life in most subjects, in accordance with other reports [1, 4]. Tendon xanthomas were diagnosed in 67% of individuals over the age of 10, while they were reported at a prevalence of 78% [1], 34% [2], 77% [3] in other cohorts. Chronic diarrhea was reported in 41% of this study and in 40% [1], 70% [2], 53% [3] of other cohorts. Seizures were observed in 35% of individuals in this study and 33% [1] and 7% [2] in other large cohort studies. Cerebellar signs were reported in 80% of individuals in this study and only 36% [1] and 39% [2] in some previous studies. Pyramidal signs were apparent in 73% of this study while previously reported at a prevalence of 64% [1] and 45% [2] in other cohorts. Osteoporosis was reported at 67% in one cohort [1] but was not reported at all in other large cohorts [2, 3]. Osteopenia or osteoporosis was reported in 27% of subjects in this study, however, there was a high percentage of individuals for whom bone mineral density was not assessed. The frequency of osteoporosis/enia among the subjects in this study for whom bone mineral density was assessed was 72%. This appears to be an underappreciated clinical feature of CTX which may actually occur at high frequency in subjects with CTX. Over nearly all clinical features of CTX there are differences in reported frequency of clinical features across various cohorts. This could be due to differences in the range of age at last report of subjects and/or may indicate various types of ascertainment bias. This could also be influenced by the fact that CTX is a multi-system disorder requiring the attention of multiple medical specialists in order to fully characterize an individual’s clinical presentation.
