# Supplementary material for: Information Theory Analysis of CTX Shows Consistent Clinical Presentation
Source: J Inherit Metab Dis. 2025 Oct 22;48(6):e70098. doi: 10.1002/jimd.70098 (PMC12541572; doi:10.1002/jimd.70098)

Supplemental Figures

Supplemental Figure 1. Flow diagram for systematic literature review

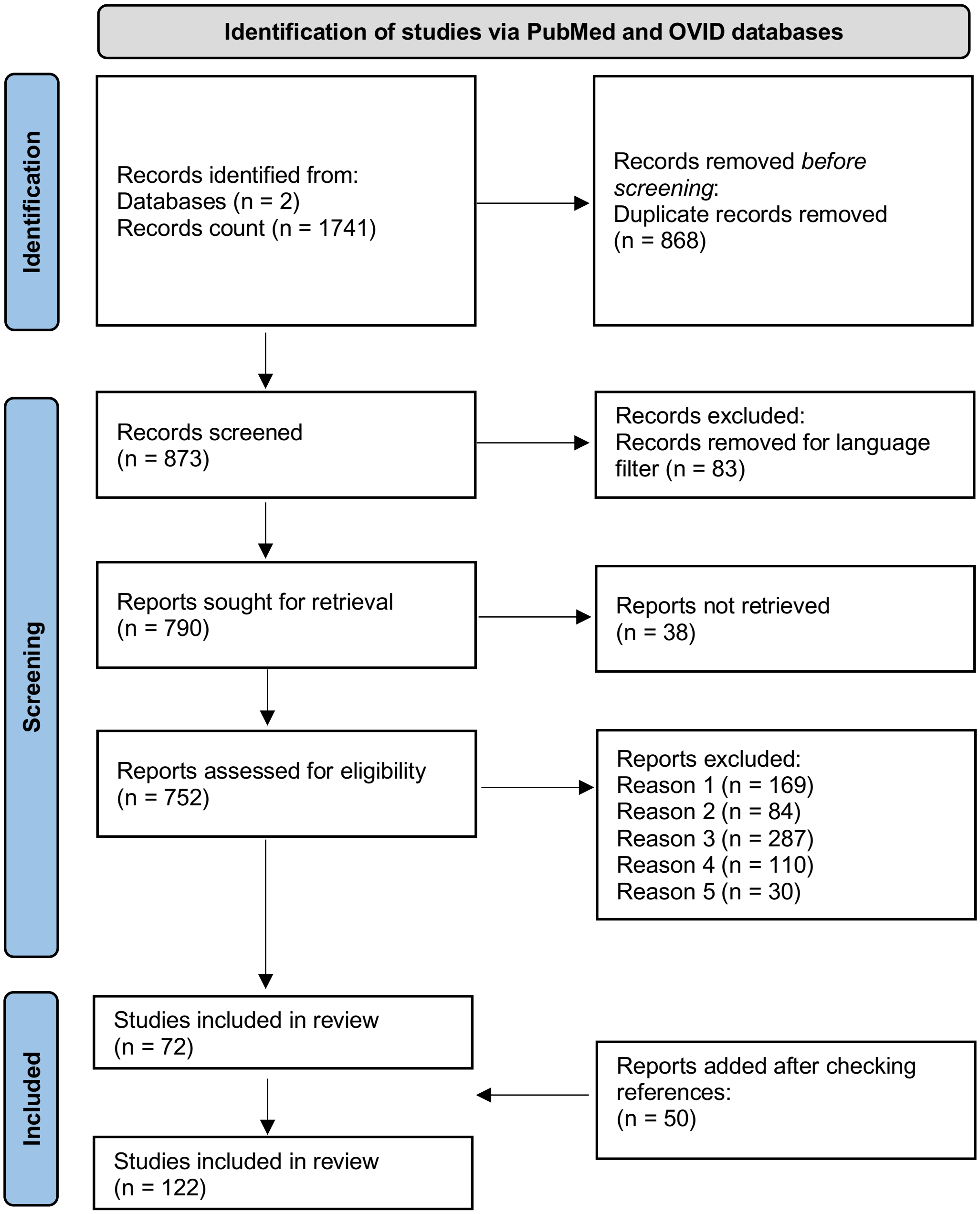

Exclusions
Reason 1 Report did not include any CTX patients
Reason 2 Report did contain clinical details for CTX patients
Reason 3 Report did not contain any families with at least 2 members with CTX
Reason 4 Report did not contain pathogenic genotype in CYP27A1 for at least 2 family members with CTX
Reason 5 Report did not contain CTX clinical details for at least 2 family members in an individually identifiable manner

Supplemental figure 2. Missing data analysis.

Supplemental figure 2A. The frequency of missing data (NR) for each clinical feature of CTX in this collection of individuals over the age of 10 years. First column is the percent of individuals in the collection of 199 subjects over the age of 10 years for whom there was no report positive or negative for the clinical feature. All subjects were reported to have or not have cataracts. The majority of subjects were not reported to have been assessed for bone density.

1	0	Cataract
2	0.03	Tendon xanthomas
3	0.70	Osteoporosis/enia
4	0.58	Intellectual disability
5	0.40	Dementia
6	0.36	Psychiatric
7	0.19	Seizures
8	0.01	Cerebellar signs
9	0.03	Pyramidal signs
10	0.42	Parkinsonism
11	0.38	Peripheral neuropathy
12	0.25	Diarrhea

Supplemental figure 2B. Missingness map for each clinical feature in this collection of individuals over the age of 10 years. Each column is a clinical feature. Each row is an individual. Red cells indicate missing data (NR). Frequency of missing data for each feature is indicated in the second row. Column labels 1-12 are shown to the right.

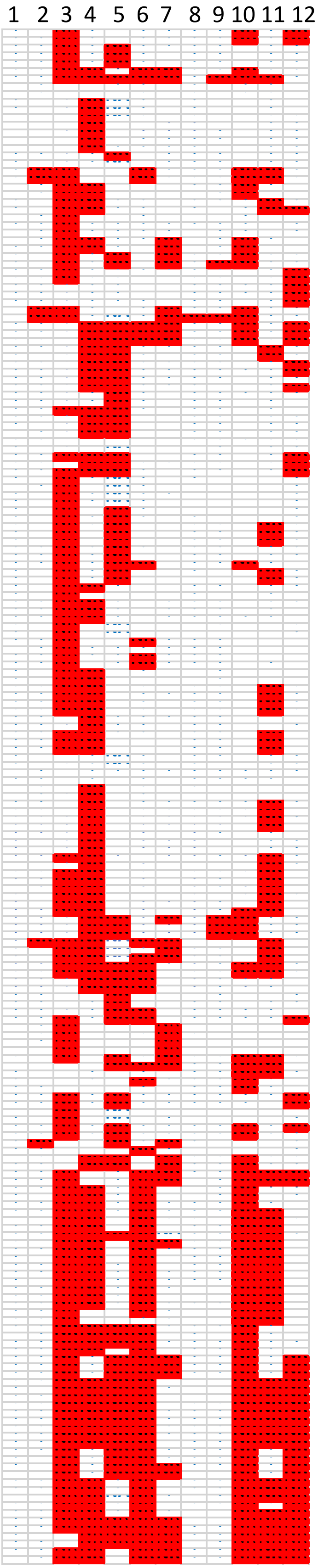

1	0	Cataract
2	0.03	Tendon xanthomas
3	0.70	Osteoporosis/enia
4	0.58	Intellectual disability
5	0.40	Dementia
6	0.36	Psychiatric
7	0.19	Seizures
8	0.01	Cerebellar signs
9	0.03	Pyramidal signs
10	0.42	Parkinsonism
11	0.38	Peripheral neuropathy
12	0.25	Diarrhea

Supplemental figure 2C. Hamming distance calculated with NR counted as a mismatch. 152 pairwise comparisons within 83 families were conducted. The distribution of Hamming distance scores calculated with NR counted as a mismatch versus the distribution of distance scores calculated with NR not counted as a mismatch was not different (Student's t-test p-value = 1).

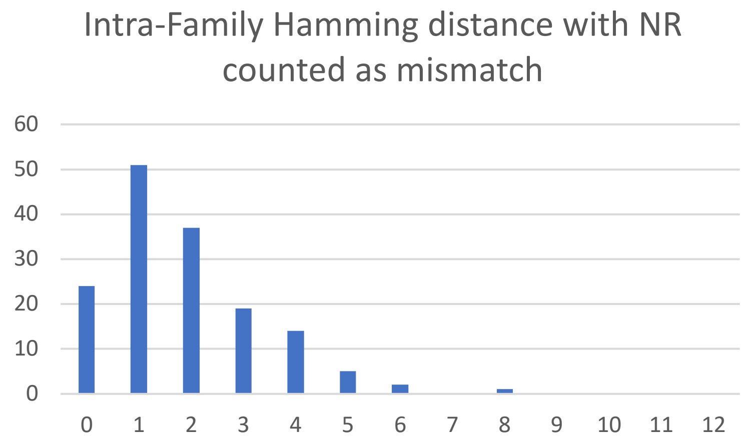

Supplement: Supplementary file 3 — Figure S1: jimd70098‐sup‐0003‐Figures.pdf. Figure S2: jimd70098‐sup‐0003‐Figures.pdf. [file JIMD-48-0-s001.pdf]
